# Pharmacokinetics and Pharmacodynamics of the Reverse Transcriptase Inhibitor Tenofovir and Prophylactic Efficacy against HIV-1 Infection

**DOI:** 10.1371/journal.pone.0040382

**Published:** 2012-07-11

**Authors:** Sulav Duwal, Christof Schütte, Max von Kleist

**Affiliations:** Department of Mathematics and Computer Science, Free University Berlin, Berlin, Germany; University of Alabama at Birmingham, United States of America

## Abstract

Antiviral pre-exposure prophylaxis (PrEP) through daily drug administration can protect healthy individuals from HIV-1 infection. While PrEP was recently approved by the FDA, the potential long-term consequences of PrEP implementation remain entirely unclear. The aim of this study is to predict the efficacy of different prophylactic strategies with the pro-drug tenofovir-disoproxil-fumarate (TDF) and to assess the sensitivity towards timing- and mode of TDF administration (daily- vs. single dose), adherence and the number of transmitted viruses. We developed a pharmacokinetic model for TDF and its active anabolite tenofovir-diphosphate (TFV-DP) and validated it with data from 4 different trials, including 4 distinct dosing regimes. Pharmacokinetics were coupled to an HIV model and viral decay following TDF mono-therapy was predicted, consistent with available data. Subsequently, a stochastic approach was used to estimate the % infections prevented by (i) daily TDF-based PrEP, (ii) one week TDF started either shortly before, or -after viral exposure and (iii) a single dose oral TDF before viral challenge (sd-PrEP). Analytical solutions were derived to assess the relation between intracellular TFV-DP concentrations and prophylactic efficacy. The predicted efficacy of TDF was limited by a slow accumulation of active compound (TFV-DP) and variable TFV-DP half-life and decreased with increasing numbers of transmitted viruses. Once daily TDF-based PrEP yielded 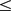
80% protection, if at least 40% of pills were taken. Sd-PrEP with 300 mg or 600 mg TDF could prevent 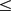
50% infections, when given at least before virus exposure. The efficacy dropped to 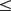
10%, when given 1 h before 24 h exposure. Efficacy could not be increased with increasing dosage or prolonged administration. Post-exposure prophylaxis poorly prevented infection. The use of drugs that accumulate more rapidly, or local application of tenofovir gel may overcome the need for drug administration long before virus exposure.

## Introduction

Tenofovir disoproxil fumerate (TDF) is an antiviral pro-drug, belonging to the class of nucleoside reverse transcriptase inhibitors (NRTIs) used for the treatment of the human immunodeficiency virus 1 (HIV-1) [1] and hepatitis B. For HIV-1 treatment, it is currently recommended as a backbone component in first-line highly active antiretroviral therapy (HAART) [2]. TDF is administered orally. After first pass of TDF through the liver, tenofovir (TFV), an analogue of the endogeneous deoxyadenosine monophosphate (dAMP) [3], is formed. TFV is also the predominant circulating form [4], [5]. After uptake into HIV target cells, TFV can become sequentially phosphorylated to form tenofovir diphosphate (TFV-DP), the active form, which is an analog of endogeneous deoxyadenosine triphosphate (dATP). TFV-DP subsequently competes with dATP for incorporation into nascent viral DNA during HIV-1 reverse transcription (RT), where it prevents further DNA polymerization during RT, once it becomes incorporated [6]. TFV-DP thus prevents the production of pro-viral DNA, which is required for stable host cell infection and viral replication.

While most studies characterize the pharmacokinetics of TFV in the blood plasma e.g. [7]–[10] only a few studies [11], [12] focus on the intracellular pharmacokinetics of the active anabolite, TFV-DP, or establish a link between the pharmacokinetics of TFV in plasma and TFV-DP in the intracellular space [13], [14], which is particularly important, since the plasma pharmacokinetics of NRTIs and the pharmacokinetics of their active intracellular anabolites are often nonlinearly related and temporally asynchronous e.g. [15], [16]. Thus, for establishing the link between dose and response, the link between plasma- and intracellular pharmacokinetics is essential, and can subsequently be used to predict the effect of drug administration on virus dynamics. This complete PK-PD link for NRTIs has only rarely been achieved [17]. For TDF, no *in silico* model exists to the authors’ knowledge, which integrates dosing, pharmacokinetics and antiviral response.

While TDF is an important drug for HIV treatment, it is also being evaluated as a core component of pre-exposure prophylaxis regimens (PrEP) to prevent HIV infection [18]. Interim reports indicate variable outcomes for PrEP strategies: Whereas some trials report no benefit of PrEP regimens (FEM-PrEP) [19], others report 44 % to 73 % reduced HIV acquisition [20]–[22]. While the efficacy of TDF-based PrEP may depend on the mode of transmission (hetero- vs. homosexual, or by needle-stick infection), it is often argued that prophylactic success could be affected by how strictly patients adhere to their (TDF-based) regimen [23]. Based on the average half life of TFV-DP in peripheral blood mononuclear cells (PBMCs) it has been previously stated that TDF is pharmacologically “forgiving” in the context of poor adherence [3]. However, TFV-DP pharmacokinetics indicate a large inter-patient variability [11], [14], potentially leading to heterogeneous protection in patients that equally adhere to their TDF-based regimen. Also, adherence in some patients in clinical trials may have been even lower than the pharmacological “forgiveness” of the drug [24].

The goal of the present study is to provide an *in silico* model that consistently predicts intracellular TFV-DP pharmacokinetics based on different TDF dosing schemes. Subsequently, we use previously published direct pharmacodynamic models to ultimately link the pharmacokinetics of oral TDF to its clinical response. Once this link is established, we use stochastic simulation to predict the relative infection risk, when TDF-based PrEP or mixed PrEP/PEP strategies are applied with different levels of adherence and timing of TDF administration and we point out factors that may impair TDF-based PrEP. In view of the recent approval of truvada (300 mg TDF + 200 mg emitricitabine (FTC)) for PrEP by the FDA, this may raise awareness, encourage experimental assessment and help to avoid the misuse of TDF-based PrEP.

## Materials and Methods

### Pharmacokinetic & Pharmacodynamic Data

TFV concentrations in blood plasma following either doses of 75, 150, 300 or 600 mg oral TDF were taken from three independent clinical trials [7], [9], [12] and used to verify pharmacokinetic model selection and evaluation (see Table S1). Individual intracellular elimination of TFV-DP was assessed using the data from [11], which observe the decline of TFV-DP in PBMCs after discontinuation of TDF treatment (see Table S2). After successful development of the pharmacokinetic model, it was coupled to a model of viral dynamics and used to predict antiviral efficacy of 28 days TDF monotherapy in asymptotically infected individuals following 75, 150, 300 or 600 mg oral TDF dosing, simultaneously estimating the PK-PD coupling parameter 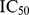 (fifty percent inhibitory concentration) and testing different alternative models for intracellular uptake and anabolism of TFV. Predicted viral load kinetics were compared to viral load data from [12] (pharmacodynamic endpoint) and used for model selection (see Text S1). The final coupled pharmacokinetic-pharmacodynamic model was used to predict the prophylactic efficacy of TDF for a wide range of parameter sets using stochastic simulation techniques.

### Assessment of Alternative Pharmacokinetic Models

We assessed different pharmacokinetic models for TFV in plasma after 75-, 150- 300- and 600 mg dosing in line with available trial data [7], [9], [12] and followed a stepwise model-building process, in which the following reasonable assumption was made: We neglected the impact of intracellular TFV-DP pharmacokinetics on the plasma pharmacokinetics of TFV, since it can be assumed to marginally influence the overall pharmacokinetics of TFV (total mass of TFV-DP in PBMCs is extremely small: 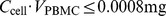; total volume of PBMCs: 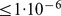
L [25], [26]; plasma volume 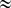
3.5L). This assumption allowed us to independently develop the plasma pharmacokinetics model and then subsequently model the influx and conversion of TFV to intracellular TFV-DP, depending on the actual TFV concentration in blood plasma.

The pharmacokinetic model building process was guided by goodness-of-fit and comparative model assessment in terms of Akaike information. Pharmacokinetic parameters were estimated by minimizing the weighted residual sum of squared errors 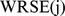 of the 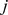
th model according to.
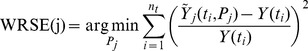
(1)were 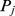 is a vector of pharmacokinetic parameters for candidate model 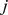, 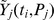 are model-predicted TFV or TFV-DP concentrations for parameter set 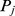 at time 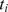 and 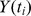 are the corresponding observed concentrations. Candidate models 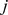 were then comparatively assessed using Akaike’s information criteria (AIC), where the AIC-value of the 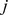
th model has been computed according to [27]:
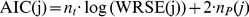
(2)where 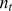 denotes the number of observations and 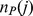 denotes the number of parameters required for the 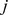
th model. Subsequently, the model with the best (the lowest) AIC was selected and further used.

### Final Pharmacokinetic Model

**Figure 1:**
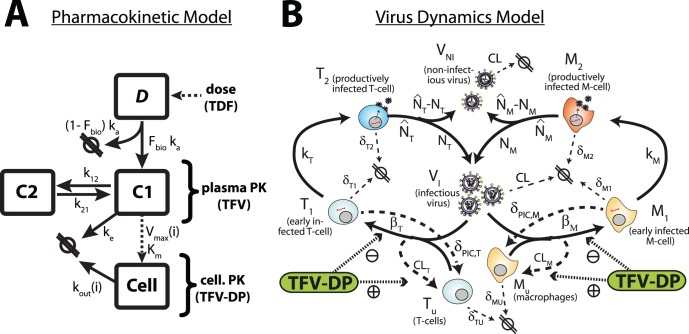
Pharmacokinetic model of TFV and intracellular TFV-DP and model of viral kinetics. A: Pharmacokinetic model. Parameters 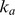 and 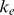 are the absorption and elimination rate constants of the central compartment **C1** (which resembles plasma pharmacokinetics of TFV) respectively. The parameters 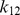 and 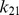 denote the influx and outflux rate constant to-/from the peripheral compartment **C2** respectively. Both compartments (central-/peripheral-) have the same volume of distribution 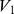. The dotted line from the central compartment to the intracellular compartment **C3** represents subsumed processes, namely the cellular uptake of TFV and subsequent phosphorylation to TFV-DP, which were related to the plasma concentration of TFV (**C1**) by Michaelis-Menten kinetics, with parameters 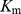 and individual parameter 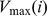. The parameter 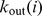 is the individual, cellular elimination rate constant of TFV-DP. B: Virus dynamics model. T-cell and macrophage target cells (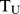, 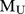) can become successfully infected by infective virus 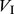 with lumped infection rate constants 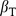 and 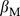, respectively, creating early infected cells 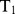 and 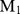. Infection can also be unsuccessful after the irreversible step of fusion (rate constant 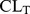 and 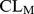, dashed lines), eliminating the virus and rendering the cell uninfected. Early infected cells 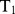 and 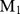 can destroy essential viral proteins or DNA prior to integration with rate constants 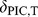 and 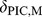 (dashed lines) returning the cell to an uninfected stage. The genomic viral DNA can become integrated with rate constants 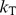 and 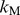 creating late infected cells 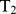 and 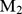, which can release new infectious- and non infectious virus 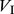 and 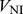 with rate constants 
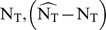
 and 
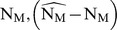
, respectively. All cellular compartments 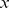 can get destroyed by the immune system with respective rate constants 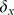 and the free virus gets cleared with rate constant 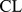 (thin dashed lines). The pharmacologically active form of tenofovir (tenofovir-diphosphate, TFV-DP, green box) inhibits successful cell-infection (parameter 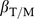) and increases the rate of unsuccessful infection (parameter 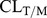).

Based on predictive performance and Akaike information (see Table 3) we found that two compartments (plus a dosing compartment) best described TFV plasma pharmacokinetics, in line with previous studies [13], [14], [28]. A third compartment was used to model the pharmacokinetics of intracellular TFV-DP [13], [14]. Intracellular pharmacokinetics of TFV-DP were linked to the plasma concentration via saturable uptake and anabolism (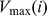 and 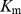) with individual maximum velocity of uptake and anabolism and individual first order elimination kinetics 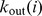 (see Table S2), which best described the available data (see Text S1). The final model for the TFV plasma- and intracellular TFV-DP pharmacokinetics is illustrated in Figure 1A. The TFV/TFV-DP pharmacokinetic model constitutes four compartments: 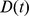 represents the mass of tenofovir in the dosing reservoir. 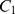 is the central compartment, which represents the plasma concentration of TFV. The second compartment 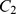 represents the poorly perfused (peripheral) tissues and the cellular compartment 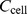 resembles the concentrations of TFV-DP in peripheral blood mononuclear cells (PBMCs). Parameters 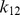 and 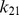 are the rate constants for influx and outflux to-/from the peripheral compartment 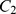 and 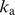 and 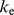 are the rates of TFV uptake and elimination into/out of 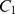 respectively. All final parameters are represented in Table 1. The value for 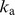 and the bioavailability 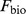 were fixed to 1 h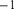 [28] and 0.32 [12] respectively, while all other parameters were estimated.

**Table 1:** Pharmacokinetic and pharmacodynamic parameters.

param.	value	param.	value
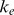	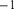 0.12 h	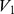	244 L
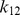	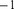 0.2926 h	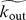	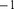 0.006 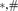 [0.002;0.026] h
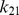	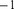 0.1537 h	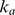	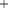 1
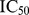	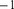 75.7 µg. L	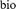 F	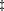 0.32
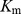	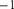 29.3 µg. L	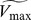	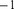 1.44 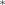 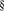 [0.5;24] µg. L 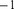 . h

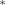
median parameter and range. 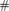 see Table 4 for individual values. 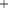 value set to 1 [28]. 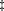 parameter from [12]. 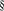 computed using eq. (S2), Text S1.

The ordinary differential equations for the final model are displayed below:
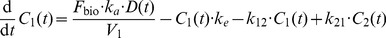
(3)

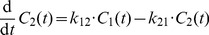
(4)

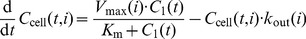
(5)where 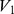 represents the volume of the central compartment. The parameters 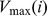 and 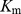 describe the (saturable) processes of TFV-uptake and conversion to TFV-DP within PBMCs, while 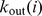 denotes the rate of elimination of TFV-DP from the PBMCs, which was found to vary between distinct patients (see Table S2 and model comparison in Text S1). The concentration in the dosing compartment 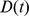 was estimated according to:
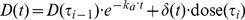
(6)where 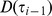 denotes the mass of TDF in the dosing compartment at the last dosing event 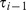. The parameter 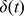 denotes a delta dirac function which takes the value 1 at the discrete dosing events 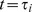 and is otherwise zero.

### Viral Dynamics

In order to predict (i) viral load kinetics following TDF treatment in HIV-infected patients and (ii) the infection probability for uninfected individuals, we adopted the virus dynamics model from [29], [30], which is depicted in Figure 1B. For predicting viral load kinetics in infected individuals, we used the deterministic infected (drug-free) fix-point of the model as a starting condition and then monitored viral dynamics following TDF monotherapy. For assessing the infection probability, we used the uninfected fix-point of the model as starting condition and inoculated the respective number of infectious viruses to simulate viral challenges.

In brief, the virus dynamics model comprises T-cells, macrophages, free non-infectious virus (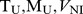, respectively), free infectious virus 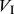, and four types of infected cells: infected T-cells and macrophages *prior* to proviral genomic integration (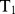 and 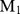, respectively) and infected T-cells and macrophages *after* proviral genomic integration (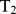 and 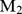, respectively). The average rates of change of the different species are given by the following system of ODEs:
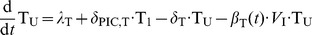


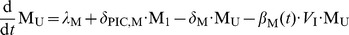


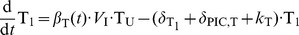


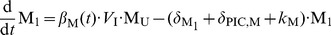
(7)

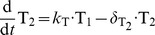


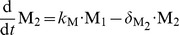


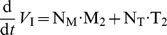


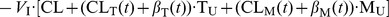


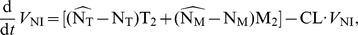
where 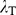 and 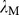 are the birth rates of uninfected T-cells and macrophages, and 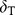 and 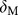 denote their death rate constants. The parameters 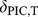 and 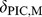 refer to the intracellular degradation of essential components of the pre-integration complex, e.g., by the host cell proteasome, which return early infected T-cells and macrophages to an uninfected stage, respectively. Parameters 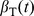 and 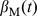 denote the rate of successful virus infection of T-cells and macrophages in the presence of TFV-DP, respectively, while the parameters 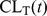 and 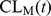 denote the clearance of virus through unsuccessful infection of T-cells and macrophages [29] in the presence of TFV-DP at the respective time 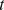. Parameters 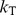 and 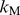 are the rate constants of proviral integration into the host cell’s genome and 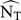 and 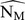 denote the total number of released infectious and non-infectious virus from late infected T-cells and macrophages and 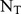 and 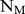 are the rates of release of infectious virus. The parameters 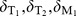 and 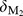 are the death rate constants of 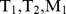 and 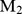 cells, respectively. The free virus (infectious and non-infectious) gets cleared by the immune system with rate constant 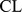.

### Pharmacokinetic-Pharmacodynamic Coupling

**Figure 2:**
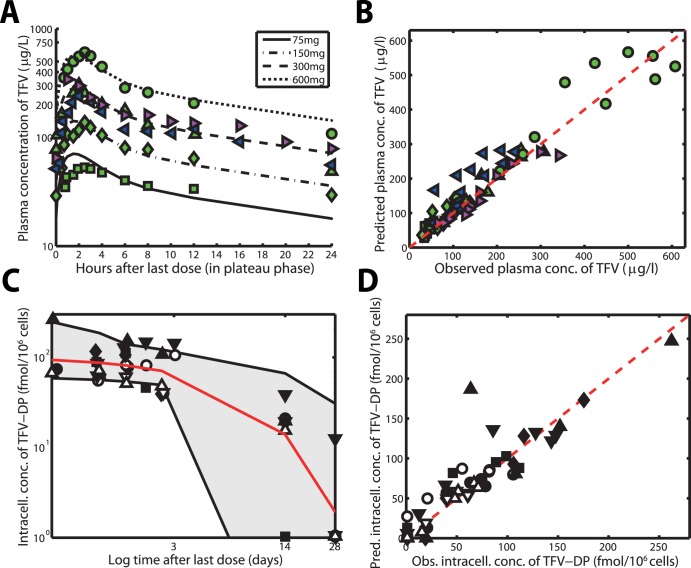
Pharmacokinetics of TFV for different doses of oral TDF at plateau and intracellular TFV-DP concentrations after treatment cessation. A: Predicted pharmacokinetics of TFV after once daily 75-, 150-, 300- and 600 mg oral TDF (lines) together with data from [7], [9], [12] (markers). B: Goodness-of-fit plot for the plasma pharmacokinetics of TFV with data from 3 clinical studies and 4 different dosing schemes [7], [9], [12]. The dashed red line indicates the line of unity, whereas the green squares, -diamonds, triangles and filled dots represent the observed TFV concentrations in [12] following 75-, 150-, 300- or 600 mg once daily administration of TDF. The blue left-pointing triangles and the magenta right-pointing triangles represent observed TFV concentrations after 300 mg once daily oral administration from [9] and [7] respectively. C: Predicted pharmacokinetics of intracellular TFV-DP after stopping of 300 mg once daily oral TDF dosing (lines) together with data from [11] (markers). D: Goodness-of-fit plot for intracellular TFV-DP. The up- and downward pointing filled and open triangles, open- and filled circles, filled squares and filled diamonds indicate intracellular TFV-DP pharmacokinetics after stopping 300 mg once daily oral TDF dosing in 8 different individuals from [11].

We have previously shown that the antiviral effect of NRTIs (like TDF) can be regarded by an inhibition of the rate of successful cell infection 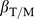 and a proportional increase in the number of unsuccessful infection events 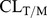 [29]. We can thus write:
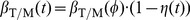
(8)

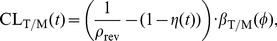
(9)where 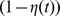 denotes the residual infection, when TFV is applied and 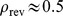 [31] is the probability that infection is successful in the absence of treatment. The efficacy of TFV-DP at time 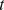 was implemented using the standard Emax-model with slope parameter 1 [32].
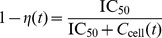
(10)where 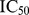 denotes the intracellular TFV-DP concentration (compartment 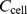 in Fig. 2A), which reduces cell infection by 50%.

### Prediction of Relative Infection Risk in the Presence of TDF

Although per-contact infection probabilities have previously been estimated for different routes of HIV transmission [33], [34] (e.g. 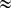
0.5–4% for homosexual receptive contact), it is not known how much infectious virus actually reaches a cellular environment that facilitates its reproduction (further on referred to as ‘inoculum size’). Moreover, several (unknown) co-factors may alter this number. It could be possible that virus does not reach a cellular environment that facilitates its reproduction during the majority of sexual contacts, as indicated by low per-contact-transmission probabilities [34]. During those sexual contacts where infection occurs, the data from [35], [36] indicate that a small number of founder particles (estimated to be of the order 1–5 in the majority of infections) establish the viral population within the newly infected individual. However, due to the inherent uncertainties about the co-factors that potentially alter the number of transmitted viruses, we will not compute relative per-contact-infection probabilities under TDF administration, but rather compute the percentage of infections prevented for distinct inoculum sizes, relative to the absence of drug. The relative infection probability is typically assessed in clinical trials from a cohort of patients, without detailed knowledge of the viral inoculum sizes and the circumstances of transmission.

In the simulations, infection was irreversible by the time that the predicted number of viruses exceeded 1 million particles (because the system behaves deterministically and approaches its infection fix-point). Therefore, we recorded an infection event during our simulations, whenever the viral population crossed this threshold in a previously uninfected ‘virtual patient’ at risk. The percentage infections prevented, when TDF is taken prophylactically was then calculated using the following formula:
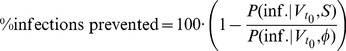
(11)where 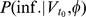 is the probability of infection in the absence of drugs 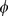, when 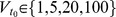 infectious viruses come into contact with a cellular environment that facilitates their reproduction within the susceptible individual. The predicted probability of infection in the absence of drugs 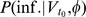 was 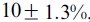, 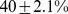, 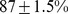 and 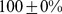, respectively, when 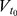 = 1, 5, 20 or 100 viruses were inoculated. 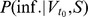 is the corresponding probability of infection when prophylactic strategy 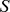 is used. We evaluated the following TDF-based prophylactic strategies: a) 300 mg oral TDF taken once daily when 20, 40, 60, 80 or 100% of pills are ingested, b) TDF is taken around the time of viral exposure (6, 1 h before exposure or 1, 6, or 48 h after exposure) and continued for 7 days (1w-PrEP/PEP) or c/d) a single oral dose of 300 or 600 mg TDF is taken at either 1, 6, 12, 24 or 48 hours before exposure to virus (sd-PrEP).

During strategy a) (once daily oral TDF) adherence was implemented using a “roulette-wheel selection” technique: A uniformly distributed random number 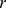 on the open interval (0,1) is drawn at each potential dosing time (each 24 hours of simulated time). If this random number 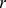 is less than or equal to the adherence level (e.g. 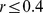 for adherence level 40%), then a dose is given to the virtual patient; otherwise not.

Modeling the infection probability requires to regard the intrinsic stochasticity and discreteness of the infection event: Either the transmitted virus becomes entirely cleared by the immune system before establishing stable infection, or the infection expands and disseminates throughout the body [18]. Reverse transcriptase inhibitors like TDF decrease the probability of cell infection and therefore increase the probability that HIV can become entirely cleared before establishing stable infection [37]. In order to fully regard the intrinsic stochasticity of rare events in the utilized model and to predict the impact of PrEP on HIV transmission, we use the stochastic-deterministic simulation algorithm presented in [30]. Unless otherwise stated, we ran 2000 stochastic-deterministic simulations for each parameter set to estimate the infection probabilities with sufficient statistical confidence.

## Results

### Plasma & Intracellular Pharmacokinetics

Predicted concentration-time profiles of TFV after 75-, 150-, 300- and 600 mg once daily dosing of TDF using the final pharmacokinetic model (eqs. (3)-(6)) are shown in Fig. 2A (lines) together with available data from 3 clinical trials [7], [9], [12] (markers). It can be seen that TFV rapidly appears in the plasma (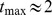 h) and decays in a bi-phasic manner for all analyzed dosing schemes. The estimated terminal half life of plasma TFV was 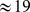 hours, in line with previous estimates [3]. TFV concentrations increase proportionally with increasing dose, indicating dose-linear pharmacokinetics. A goodness-of-fit plot with regard to plasma concentrations is shown in Fig. 2B. The plot indicates an overall spread around the line of unity, supporting the predictive power of the model. The predicted decay behavior of TFV-DP in PBMCs after stopping TDF dosing is shown in Fig. 2C together with available data [11]. The grey area therein indicates the predicted range of kinetic behavior, whereas the solid red line indicates the estimated median TFV-DP decay. Note that the variation (grey range) is quite large, which is however in line with other studies [14]. A goodness-of-fit plot with regard to individual predicted vs. observed intracellular TFV-DP concentrations is shown in Fig. 2D for the data coming from the distinct patients (markers). The predicted average half life of TFV-DP was very large 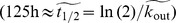. Overall, the plot indicates a spread around the line of unity (dashed red line in Fig. 2D), supporting the approach chosen for estimating individual decay kinetics of TFV-DP in peripheral blood mononuclear cells (PMBCs) rather than using an average value for all patients (see also model comparison in Text S1).

**Figure 3:**
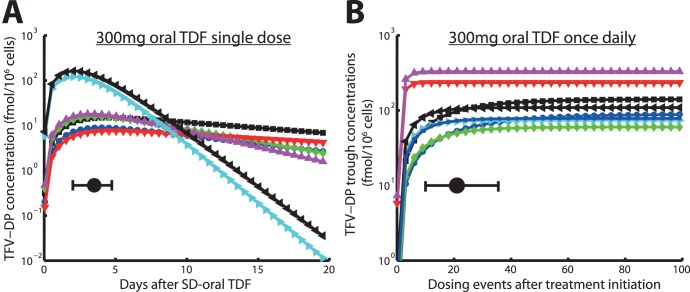
Predicted TFV-DP intracellular pharmacokinetics following a single dose oral 300 mg TDF and accumulation of TFV-DP after daily 300 mg oral TDF. A: Predicted intracellular pharmacokinetics of TFV-DP in PBMCs after a single 300 mg oral TDF dose. Solid black circle and horizontal error bar indicate the 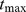 value and its range. B: Trough levels of TFV-DP in PBMCs following 300 mg oral TDF every 24hours, indicating the accumulation of active compound. The solid black circle and the horizontal error bar indicate the time until plateau concentrations are reached and the range for this parameter. Blue cirles, black squares, green diamonds, red downward pointing triangles, magenta upward-pointing triangles, cyan right-ward pointing triangles, black left-pointing triangles and blue asterisks indicate individual predictions for 8 patients.

The predicted concentration time profile of TFV-DP after a single dose of 300 mg oral TDF is shown in Fig. 3A. It can be seen that TFV-DP reaches its maximal concentrations after a median time of 85 h (range: 49–113 h) following a single dose of TDF. The maximally achievable concentrations vary between individuals and are within the range of 7.6 to 163 fmol/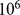 cells (median value: 16 fmol/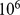 cells) in case of a single dosing event.

The accumulation of intracellular TFV-DP in the case of daily 300 mg oral TDF is shown in Fig. 3B. TFV-DP trough concentrations (concentrations immediately before the next dose) reach their plateau levels after a median of 21 once daily dosing events (range 10–36). On the contrary, plateau levels of the parent compound TFV are reached within 7 dosing events in blood plasma already (data not shown).

### Antiviral Efficacy During Mono-therapy in HIV-infected Individuals

**Figure 4:**
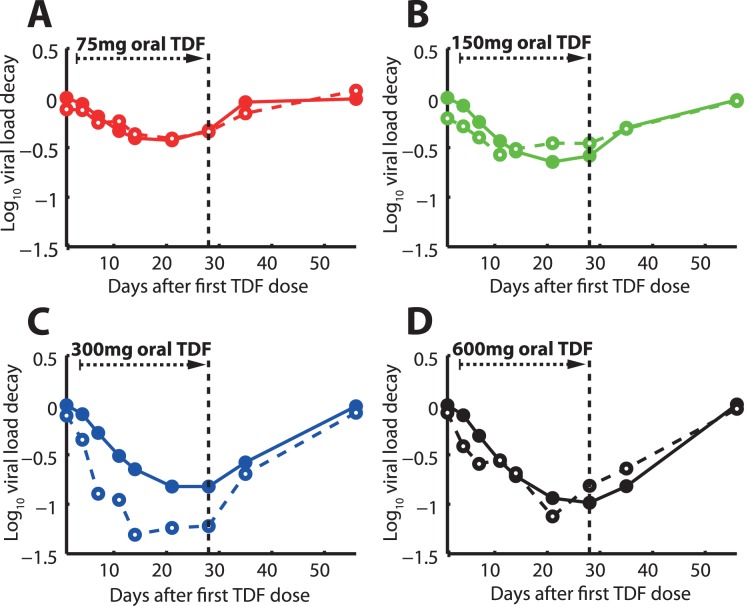
Viral load log_10_ kinetics during- and after 28 days of TDF mono-therapy. Black dashed vertical lines indicate the withdrawal of TDF dosing. Solid lines represent predicted median viral kinetics using the coupled PK-PD model, whereas dashed lines represent the observed viral kinetics [12]. Once daily 75mg TDF dosing. B: Once daily 150 mg TDF dosing. C: Once daily 300 mg TDF dosing. D: Once daily 600 mg TDF dosing.

For further model evaluation and estimation of the remaining parameters 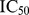 and 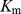, we coupled the pharmacokinetics of intracellular TFV-DP to an established model of the HIV-life cycle [28], [30] (see *Methods* section) and subsequently predicted the 56 days viral dynamics in asymptomatically HIV infected individuals following a 28 days mono-therapy (day 0–28) with either 75-, 150-, 300- and 600 mg TDF. Our predictions are shown in Fig. 4A, B, C, D together with data from the corresponding dose escalation study [12]. The dashed lines and open circles in Fig. 4A, B, C, D indicate clinically observed median log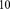 viral load decay from [12], whereas the solid lines and filled circles indicate the predicted median log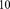 viral decay using our model. The respective weighted residual sum of squared errors WRSE (see eq. (1)), denoting the absolute deviation between experimental and predicted viral load decay is shown in Text S1 (the Table therein) and indicates an overall good predictive power of the coupled PK-PD model. Notably, the experimental median viral decay profile for the 300 mg dose group indicated maximally achievable viral decay, as the 600 mg dosing could not produce steeper viral decay than the 300 mg scheme.

### Efficacy of Daily TDF for the Prevention of HIV-1 Infection

**Figure 5:**
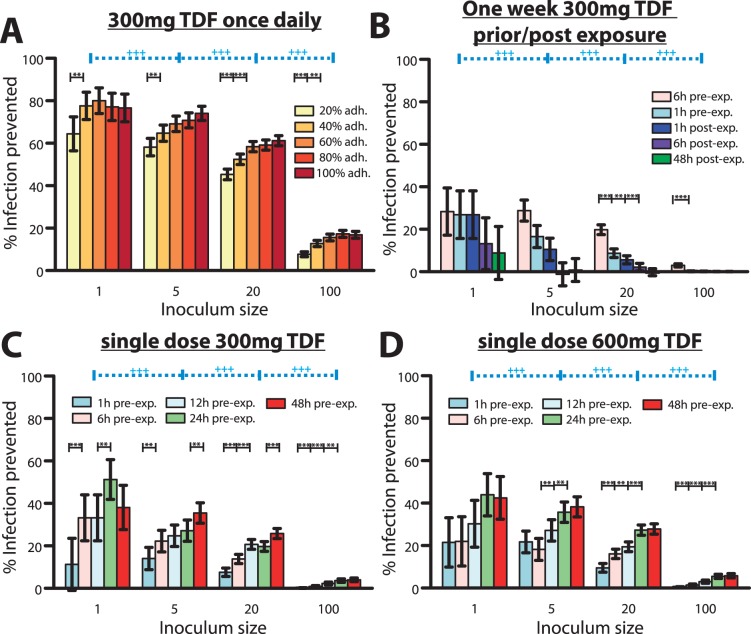
Predicted % infections prevented by distinct TDF-based prophylactic strategies for various parameter sets. A: Predicted % infections prevented by once daily 300 mg TDF taken at different levels of adherence and with distinct virus inoculum sizes. 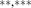 prophylactic efficacy depends on adherence at the p 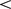 0.05 or p 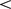 0.01 level respectively. B: Predicted % infections prevented by a one week 300 mg TDF (1w-PrEP/PEP) when started at distinct times before/after exposure with distinct numbers of viruses. 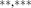 prophylactic efficacy depends on the timing of start of TDF administration at the p 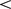 0.05 or p 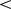 0.01 level respectively. C: Predicted % infections prevented by a single dose 300 mg TDF (sd-PrEP) when taken at distinct times before exposure with distinct virus inoculum sizes. 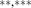 prophylactic efficacy depends on the timing of TDF single dose administration at the p 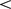 0.05 or p 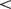 0.01 level respectively. D: Predicted % infections prevented by a single dose 600 mg TDF (sd-PrEP) when taken at distinct times before exposure with distinct virus inoculum sizes. Error bars represent confidence bounds calculated using Greenwood’s formula. 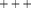 prophylactic efficacy depends on the inoculum size. The predicted probability of infection in the absence of drugs 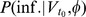 was 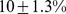, 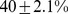, 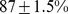 and 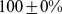 when 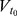 = 1, 5, 20 or 100, respectively, viruses were inoculated.

The predicted percent infections prevented by continuous once daily 300 mg TDF PrEP are shown in Fig. 5A. It can be seen that continuous PrEP can avert 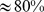 infections, under 100% adherence and with small inoculum sizes (1 infectious virus). Under a fivefold increase in inoculum size, TDF is still efficacious, preventing 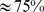 of infections. However, if the inoculum size is further increased (100 infectious viruses come into contact with target cells), the efficacy drastically drops to levels of 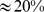 protection. On the other hand, imperfect adherence above the level of 40% has only a small impact on the predicted efficaciousness of TDF, confirming previous pharmacologic considerations about the pharmacokinetic forgiveness of the drug [3]. We statistically tested whether adherence and inoculum size impact on the efficacy of TDF-based PrEP, based on our simulation results. We found that decreasing adherence has a small impact of the efficacy of TDF-based PrEP (infection probabilities are not significantly altered if adherence is as low as 60%). However, if adherence is below 40%, TDF-based protection is significantly altered (p 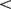 0.05). Furthermore, when large numbers of viruses become transmitted, we observe a stronger impact of adherence (see Fig. 5A). The inoculum size determined the efficacy of TDF-based PrEP for all conditions tested (p 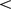 0.01, see Table S3).

In summary, the protective effect of TDF appears to be much less sensitive to poor adherence (as long as adherence is above 40%), but is dependent on the actual mode of transmission, i.e. how many viruses become transmitted. Notably, in a substudy of Partners PrEP (serodiscordant couples in Kenya/Uganda) using TDF only, an overall efficacy of 62% (confidence interval: 34%;78%) was reported, which corresponds to our predictions for the case when small numbers of viruses become transmitted (inoculum size 1–20 in Fig. 5A). The number of distinct founder viruses was estimated to be rather low (of the order 1–5 for heterosexual- and homosexual transmission) [35], [36], which stresses the importance of PrEP efficacy at low inoculum sizes for the prevention of HIV-1 transmission and supports the predictive power of our model.

### Efficacy of One Week extended TDF prophylaxis during Viral Exposure

We predicted the efficiency of TDF when started either 6 or 1 h before exposure or 1, 6, or 48 h after exposure and continued for 7 days (1w-PrEdP/PEP). The results (see Fig. 5B) indicate a maximally achievable efficacy of 1w-PrEP/PEP of 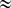
30% when started 6h before viral challenge for small inoculum sizes. The maximum achievable efficacy was similar to the sd-PrEP regimen. The efficacy of 1w-PrEP/PEP was influenced by inoculum size (p 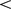 0.01, for all tested conditions, see Table 6) and dropped drastically as the inoculum size increased. 1w-PrEP/PEP efficacy was also affected by the timing of TDF initiation, particularly for large inoculum sizes (p 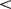 0.05, see Fig. 5B), with earlier times of regimen initiation resulting in higher efficacy. Overall, our predictions indicate that extended (one week) prophylaxis with TDF initiated shortly before viral exposure offers little benefit compared to sd-PrEP (Figure 5C, D). If TDF is initiated after viral exposure, its efficacy is rather limited.

A recent investigation showed that 28 days of a post-exposure prophylactic triple drug regimen containing TDF [38]–[40] is safe, but data indicating the efficiency is missing for TDF alone or TDF containing regimen in humans. Efficacy of PEP using tenofovir has to date only been demonstrated in non-human studies, e.g. [41], [42]. The conducted experiments, however, indicate that the prophylactic efficacy of post-exposure TDF may depend on the type of virus used [43] and on particular pharmacokinetics, possibly limiting the translation of these results to TDF-based PEP in human.

### Efficacy of Single Dose TDF Prophylaxis Shortly before Exposure (sd-PrEP)

We tested the efficacy of single dose 300- and 600 mg oral TDF given either 1, 6, 12, 24 or 48 h before viral exposure in Fig. 5C, D respectively. Notably, sd-PrEP could reach a maximum efficacy of 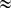
50% with small inoculum sizes, when given 24 hours prior to exposure. The efficacy dropped gradually when the inoculum size increased. In particular, sd-PrEP was completely inefficient when large inoculum sizes were encountered (if 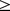 100 infectious viruses come into contact with target cells). The dependency of sd-PrEP efficacy on inoculum size was significant for all tested conditions at the p 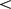 0.01 level (Table S5, S6). Despite a dependency on the inoculum size, sd-PrEP efficacy was also significantly altered by the timing of drug administration, see Figure 5C, D. Generally speaking, sd-PrEP efficacy was highest if TDF was taken 12–48 h before viral exposure and almost completely inefficient when taken only 1 h before exposure, which limits it’s practical use as a single-dose prevention drug. The poor efficacy of sd-PrEP, as well as the dependency on the timing of TDF administration is based on its pharmacokinetics: TFV-DP, the active moiety, requires approximately 21 (range: 10–36, see Fig. 3B) dosing events to reach plateau levels and to exert its maximum effect. During single dose administration, TFV-DP still requires about 85 h hours (range: 49–113, see Fig. 3A) to reach maximum concentrations 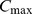. Therefore, TDF needs to be taken early enough (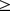 48 hours) to allow for intracellular TFV-DP levels to build up. Once TFV-DP levels have been achieved, they persist in most patients, owing to the long half life of intracellular TFV-DP.

We also tested whether the effect of single dose TDF PrEP could be potentiated, if the standard dose was doubled (see Figure 5D). The prophylactic efficacy was, however, not markedly different for most conditions tested, see Fig. 5C&D and Table S7 for a statistical evaluation.

### Relation between Intracellular TFV-DP Concentrations and Prevention of HIV-1 Infection

**Figure 6:**
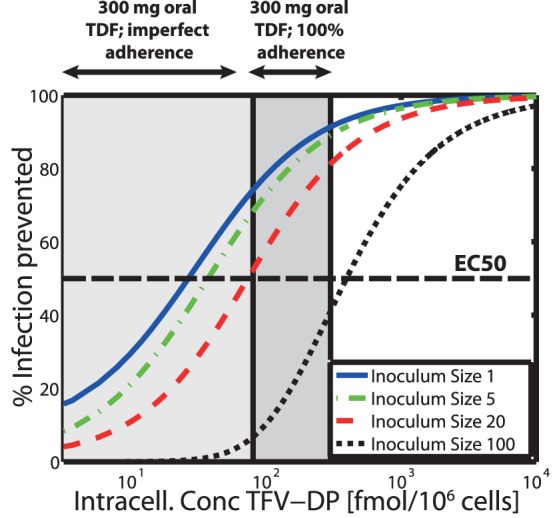
Predicted % infections prevented vs. intracellular TFV-DP concentrations for distinct virus inoculum sizes. The solid blue-, dash-dotted green, dashed red and dotted black lines show the concentration-response profile for virus inoculum size 1, 5, 20 and 100 respectively. The thick dashed horizontal black line indicates the TFV-DP concentration, which prevents 50% of infections (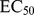). The dark grey area indicates the TFV-DP concentration range achieved during once daily 300 mg oral TDF dosing with 100% adherence, whereas the light grey extension to the left indicates the range of concentrations resulting from imperfect adherence. Predictions are based on the approximate analytic solution derived in Text S2.

We have derived an analytical formula in Text S2 to assess the relation between intracellular TFV-DP concentrations and the % HIV-1 infections prevented. The percent infections prevented by distinct intracellular TFV-DP concentrations is shown in Fig. 6 (based on the analytic solution). It can be seen that the 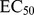 value (concentrations of intracellular TFV-DP necessary to prevent 50% of HIV-1 infections) is increasing for larger virus inoculum sizes. The computed 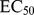 values were 29, 40, 77 fmol/10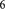 cells for inoculum size 1, 5 and 20 respectively, which is below the concentration range achieved when 300 mg TDF is given once daily in an adherent patient (dark grey area in Fig. 6). On the contrary, the 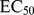 for a viral inoculum size of 100 is above the concentration range typically achieved during once daily PrEP with 300 mg TDF (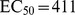 fmol/10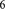 cells). TFV-DP concentrations to prevent 90% infections 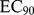 were 267, 348, 640 and 2866 fmol/10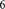 cells for virus inoculum size 1, 5, 20 and 100 respectively (see Fig. 6).

## Discussion

The plasma pharmacokinetics of TFV were best described by a two compartment model (compartments 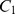 and 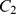) with first order absorption and elimination, based on statistical model comparison. Similar models were also used by most other groups to describe the pharmacokinetics of TFV in blood plasma [14], [28], [44], [45]. Pharmacokinetic parameter estimates (Table 1) agree well with previous studies [14], indicating a large volume of distribution, bi-phasic decay with a particularly slow terminal half life of 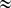
19 h, in line with previous estimates [3]. Inter-individual variations in parameter values characterizing plasma pharmacokinetics were estimated to be small in related studies [14], [28], [44] (coefficient of variation less than 50%). We therefore decided to ignore inter-individual variations in parameters describing the plasma pharmacokinetics of TFV. To the contrary, parameters describing the intracellular pharmacokinetics of TFV-DP display a large inter-individual variability (in our model this affects parameters 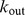 and 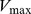).

NRTIs like tenofovir exert their effects through their intracellular phosphorylated moieties, which are often non-linearly related to plasma pro-drug concentrations [15], [17], [46]. As a consequence, plasma pro-drug concentrations may poorly predict pharmacological activity [47], [48]. For NRTIs it is therefore necessary to model the pharmacokinetics of the active intracellular form explicitly. Here, we followed a step-wise model building process to establish the link between plasma pro-drug and intracellular TFV-DP pharmacokinetics, where we first independently estimated intracellular TFV-DP elimination. Statistic model evaluation using typical- vs. individual estimates of the elimination rate constant 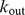 indicated that taking intracellular pharmacokinetic variations into account does not only improve the prediction of intracellular TFV-DP concentrations (see Fig. 2D), but also improves the prediction of viral decay following TDF mono-therapy with different doses (see Text S1). Notably, we predicted a large variation for the 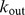 parameter (range: 0.002–0.026 h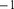), which is, however, within the confidence interval of previous estimates (confidence interval: 0.0007–0.0372 h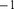) [14]. The typical half life of TFV-DP was very large (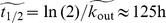; range: 26–386h), which is in good agreement with other studies [14], [49], [50]. Due to the lack of intracellular TFV-DP pharmacokinetic data illuminating the uptake of this specimen, we estimated the kinetics of influx/anablism of intracellular TFV-DP (and the 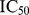 value) by comparing viral decay kinetics following 28 days of TDF mono-therapy with different doses. Based on model comparison, we found that a saturable influx with individual (maximally achievable) influx rates would best describe the pharmacodynamic data. Notably, others [13], [14] also found a saturable uptake based on pharmacokinetic data alone (without taking viral decay into account) and found a large variation in the uptake rate [14], consistent with our findings. The saturable uptake kinetics translate into maximally achievable TFV-DP concentrations, which results in maximally achievable viral decay upon increasing doses of TDF. As can be seen in Fig. 4C & D (dashed lines) clinically measured viral decay from [12] appears to be greater for 300- vs. 600 mg TDF, which was not reproduced by our model predictions (solid lines in Fig. 4C & D). The authors of the clinical report [12] however stated that the difference in viral decay between the two doses was not significant and may be attributed to noise and the small size of the population tested (8 individuals for each dose respectively in [12]) rather than having a mechanistic reason.

In previous studies, average plateau TFV-DP concentrations from different studies were in the range 80 to 160 fmol/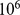
cells [3], [14], whereas the individual TFV-DP concentrations varied between 10.6 to 441 fmol/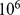
cells [14] when 300 mg oral TDF was administered once daily. Our model predicted average plateau levels were 130 fmol/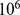
cells (range: 52–327 fmol/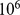
cells; see Fig. 3B), which is consistent with previous findings. TFV-DP accumulates very slowly, owing to its long half life. We estimated that plateau concentrations will be achieved after 21 dosing events (range: 10–36), which is in the range of previous pharmacologic considerations [3] (23 once daily dosing events). The slow accumulation of TFV-DP limits its prophylactic use as a single dose drug, although prophylactically effective concentrations may already be achieved 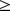 24h after a single dosing event in some patients (see Fig. 3A). In the absence of data reporting TFV-DP concentrations in PBMCs after a single 300 mg oral TDF dose we are, however, not able to directly verify these predictions. Notably, very similar TFV-DP concentrations in rectal tissue biopsies after a single 300 mg oral TDF dosing event were observed by Patterson et al. in a very recent study [51] (discussed later on).

As suggested by Piliero et al. [47], the intracellular half life of phosphorylated NRTIs is a key determinant of their clinical efficacy. Often, however, the typical half life from different individuals is taken as a reference and inter-individual differences in the pharmacokinetics of activated NRTI anabolites are neglected. In the case of TDF, large variations in the intracellular pharmacokinetics may exist, which warrant further investigation in order to optimize its efficacy both for prophylaxis and treatment.

We predicted that the long half life of intracellular TFV-DP translates into desirable properties in the case of continuous PrEP, which is pharmacologically ‘forgiving’ in the case of poor adherence, if at least 40% of the pills are ingested (see Fig. 5A). While these pharmacologic considerations have been previously discussed [3], we are presenting a quantification of these effects by combining pharmacokinetics, viral dynamics and stochastic simulation in a single integrated *in silico* model.

It was recently suggested that the willingness to take pills may be a major obstacle for the implementation of PrEP strategies in practice [24]. In line with this statement, Donnel et al. [52] found a significant difference in HIV infection between individuals with detectable vs. undetectable TFV in blood. Of note, for the levels to drop from 70 ng/mL (median concentrations in [52]) to 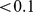 ng/mL (limit of detection in [52]), patients require to take less than 14% of their drugs (one out of seven doses), as TFV exhibits a long terminal half life in plasma (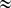
19 h). This indicates that the willingness to take daily medication for HIV prevention may be extremely low in some individuals with undetectable drug (adherence 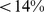). It also raises concern that willingness to take PrEP may in fact be a major obstacle for the implementation of PrEP in practice as considered by Van Damme et al. [24]. The results by Donnel et al. [52] and Van Damme et al. [24] also indicate and that the estimates of PrEP efficacy may have been contaminated by extremely poor adherence of some individuals in the trials. In agreement with this assumption, clinical outcomes with TDF-based continuous PrEP indicate highly variable outcomes: from either being inefficient (FEM-PrEP) [19] to 44–73% reduced HIV acquisition [20]–[22]. A sub-study of Partners PrEP assessed the efficacy of continuous 300 mg daily TDF administered to the healthy partner in sero-discordant couples in Kenya and Uganda. The overall efficacy was 62% (confidence interval: 34%;78%) and may be higher in adherent patients [24]. We predicted a prophylactic efficacy of 65%–80% for inoculum size 1–5 in patients that take at least 40% of their drugs, see Fig. 5A. In view of the possible contamination of Partner PrEP trials results by extremely poor adherence in some individuals, our slight overprediction of TDF efficacy may be anticipated. Further analysis is required in order to assess the proportion of individuals with *sufficient* adherence.

In the case of short-course pre-exposure TDF, or post-exposure TDF, prophylactic success is limited by a slow accumulation of the intracellular active component TFV-DP (only 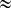
20% infections are prevented if TDF is taken 1h before exposure, see Fig. 5B, C, D and Table S4, S5, S6. Note also that intracellular TFV-DP may require 21 dosing events on average to reach plateau levels, see Fig. 0B. In view of the recent approval of Truvada (300 mg TDF +200 mg emtricitabine) for pre-exposure prophylaxis by the FDA, prescribers should inform their patients about these potential shortcomings, in order to avoid HIV-1 infection by inadequate use of prophylaxis in combination with risk compensation [53]. HIV-infection in combination with the inadequate use of PrEP may also select drug resistance, which could limit treatment perspectives for infected individuals. In terms of short-course pre-exposure prophylaxis other drugs may be more suitable that accumulate rapidly, such as nevirapine [37], which is successfully used for prevention of mother-to-child infection.

Based on the model parameters, the duration of action required to ensure that virus particles are eliminated with e.g. 99% probability, 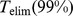 may be computed according to 
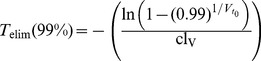
, with cl_v_ = CL+*β*_T_(*t*)⋅T_U_+*β*_M_(*t*)⋅M_U_. For the parameters used 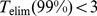 days would suffice for inoculum sizes 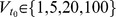. Taken together, this may indicate that, *pharmacologically*, single dose PrEP drugs taken shortly before potential viral exposure are required to accumulate rapidly in target cells, but may not have to persist for more than 3 days, in line with the pharmacological attributes of most NNRTIs.

In contrast to our predictions (Fig. 5B), some non-human studies found that TDF-based post-exposure prophylaxis may be highly efficient: Tsai et al. [42] treated macaques for variable durations after exposure with SIVmne and tested viral markers. In their non-human model of TDF-based PEP, viral titers remained undetectable in some monkeys until week 48 post-exposure, indicating that some protection was achieved, in particular for longer durations of PEP (28 days) and timely start of prophylaxis (within 24 hours post-exposure) [42]. It was however argued [54] that TDF-PEP may enhance immune controlled viral replication down to undetectable levels, rather than actually preventing infection. Furthermore, the efficacy in the primate model were depending on the type of virus used [43] (which are SIV strains, not HIV-1) and may also depend on the particular pharmacokinetics in the primate model, which may be different to the human. Altogether, the non-human studies with TDF-based PrEP may not translate into human.

It is not precisely known how much virus is being transmitted from an infected to an uninfected individual during e.g. sexual contact. Moreover, it is not known how many transmitted viruses actually reach a target cellular environment that allows their reproduction, and what types of cells are relevant for the initial infection. Also, the number of transmitted viruses, the availability of target cells and the subset of viruses that reach a cellular environment that facilitates their reproduction may be altered by the circumstances of HIV-1 transmission and several unknown co-factors. While the earliest stages of mucosal transmission of HIV-1 have not been directly observed in human and are not fully understood, animal experiments suggest that CD4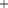 T-cells are probably the principal cell type infected at the portal of entry and throughout the earliest stages of infection [55]. These cells are mainly located in the sub-mucosa [56]. Although exposure at the mucosal surface may be substantial, only a fraction of HIV-particles may penetrate the intact epithelial layer and reach target cells [57]–[59] (denoted as inoculum size throughout the manuscript). Low per-contact infection probabilities further indicate that infectious virus may not reach a cellular environment that facilitates their reproduction during most sexual contacts [33], [34] (per-contact infection probabilities 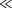
5%), in contrast to other routes of transmission such as blood transfusion [34] (per-contact infection probabilities 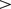
95%). Recent studies further showed, based on genotyping, that most infections (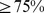) resulting from sexual HIV-1 transmission can be traced back to a single founder virus, or small populations of founder viruses [35], [36]. Since the majority of new infections result from sexual HIV-1 transmission, PrEP intervention strategies may already effectively curb sexual HIV-1 transmission by preventing infection with small virus inoculum sizes. However, in the presence of co-existing infections, the integrity of the mucosal barrier may be compromised, which increases inoculum size [60]. Furthermore, co-existing infections may increase HIV-1 acquisition by increasing the availability of target cells in the sub-mucosa [60]. While we did not take co-infections into account, future research is warranted to elucidate the role of co-infections in the context of PrEP-strategies.

Our predictions revealed that the prophylactic efficacy of TDF decreases with an increasing number of inoculated viruses (see Fig. 5A, B, C, D), making TDF more efficient when only a few viruses reach a target cell environment and less efficient for large numbers of viruses. This observation can be explained as follows: a) At clinically relevant concentrations, TDF may only inhibit a certain proportion of potential target cell infections 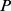. b) Some minimum number of infectious viruses 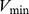 may already result in infection with almost 100% probability. When only a proportion of potential target cell infections are prevented, some inoculum size 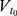 exists where 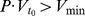. Therefore, TDF becomes inefficient above a certain inoculum size. The effect of TDF is particularly limiting, if 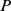 cannot be decreased by increasing TDF dosage (TFV uptake & anabolism become saturated, see eq. (5) and grey range in Fig. 6).

While it has recently been suggested to combine antiviral strategies for HIV-1 prevention [61], in this work, we predict a dependency of PrEP efficacy on inoculum size, which could make combined HIV prevention efforts synergistic: ‘test and treat’/’treatment as prevention’ strategies [62] aim to reduce the infectiousness of seropositive individuals by initiating HAART immediately after diagnosis, which effectively down-sizes their viral load and therefore the number of viruses transmitted to an uninfected individual. We predict that PrEP is highly efficient in the scenario where only few viral particles become transmitted, which possibly makes the two HIV-prevention efforts synergistic. This assumption, however, warrants further experimental confirmation.

The developed model is based on several assumptions, which we outline in the following:

- We used intracellular TFV-DP concentrations in PBMCs as a marker of efficacy. PBMCs are surrogate markers, which consist of different cell types of which the majority, however, is susceptible to HIV-1 infection [26]. Different cell types may differentially phosphorylate TFV, depending on the expression of transporters and enzymes relevant to the intracellular phosphorylation of this drug. In line with this argument, Patterson et al. [51] recently found higher levels of TFV-DP in tissue biopsies from the rectum as compared to cervix and vagina after a single dose of Truvada (300 mg TDF and 200 mg emtricitabine). Remarkably, the median concentrations of TFV-DP in the rectal biopsies (displayed in units fmol/g tissue in [51]) are within the same range as those concentrations predicted in Figure 3A after unit conversion (1 fmol/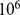 cells 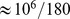 fmol/mL tissue; 1 mg tissue 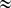
1 mL tissue). However, it is not entirely clear, what implications the distinct TFV-DP levels detected by Patterson et al. [51] may have in terms of HIV-1 prophylaxis: Only a subset of cells in the genital/rectal biopsies may be relevant for HIV-1 infection (e.g. CD4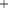 lymphocytes [55]). Thus, it is not entirely clear if e.g. lower TFV-DP concentrations in these biopsies imply lower concentrations in cells *relevant* to HIV-1 infection or only in those *not relevant* to infection. Human studies, which analyze TFV-DP levels in purified CD4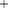 cells, are missing. Purified CD4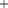 cells derived from rectal biopsies in macaques indicate identical TFV-DP levels when compared to PBMC levels, which suggests that the PBMC surrogate marker is a good indicator for TFV-DP levels in cells relevant to HIV-1 infection.
- Recent work suggests that the efficacy of NRTIs like TDF is affected by the levels of endogenous competing nucleotides dNTP (specifically: dATP for TFV-DP) [6], [63]. Although this is likely to contribute to the efficacy of TFV-DP to prevent particular routes of infection, we could not take this information into account, because information concerning dNTP levels in target cells in different physiologic locations is lacking for humans. However, once these levels become available, their impact on the (cell-specific) susceptibility may be probed by sophisticated models, such as [6].
- Vaginal TFV gel has been used successfully to prevent heterosexual HIV-1 infection [64]. Vaginal TFV gel exhibits entirely different pharmacokinetics compared to oral TDF dosing. TFV-DP levels in vaginal lymphocytes may be significantly higher in relation to the systemic levels (TFV-DP in PBMCs) after local exposure [65], [66]. Most importantly, local exposure may mitigate the need for dosing long before exposure, which may be the greatest obstacle for the sucess of oral PrEP in practice. While the current model is useful in predicting the effects of oral TDF administration on HIV-1 infection, sophisticated pharmacokinetic modelling of vaginal TFV gel [67] in combination with stochastic modelling may enable to assess its prophylactic efficacy *in silico* in the future.
- In the absence of data elucidating the levels of TFV-DP in uninfected individuals, we assumed that TFV-DP levels in PBMCs from infected individuals vs. uninfected individuals are similar. Since TFV pharmacokinetics (parent compound) have been reported to be similar in healthy- and HIV-infected individuals [5], we found it reasonable to assume that TFV-DP levels are also similar.
- It has recently been reported that TFV may become phosphorylated within red blood cells (RBCs) [68]. While standard procedures for the preparation of PBMC samples may not prevent their contamination with RBCs, this may hamper the accuracy of determination of TFV-DP in PBMC samples. Therefore, differences in RBC contamination may in part contribute to the variability of TFV-DP levels in PBMC measurements. The relevance and impact of RBC contamination on TFV-DP levels is not yet fully understood and further research is warranted to assess its role.
- In individuals with established infection, the rates of viral elimination 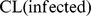 have been determined in a number of clinical studies, see e.g. [69], [70]. Because of ethical reasons, the elimination of HIV in uninfected/newly infected individuals 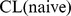 has never been directly observed. We assumed that viral elimination 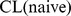 is lower in uninfected than in infected individuals, because the immune system may not recognize HIV readily in the naive patient. In line with other studies [71], [72], we therefore set the parameter 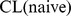 = 2.3 [1/day] (see Table 2), which reproduced clinical infection probabilities in previous work [37].

**Table 2:** Parameters used for the viral model.

Param.	Value	Ref.	Param.	Value	Ref.
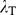	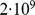	[73]	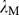	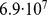	[74]
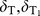	0.02	[74]	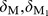	0.0069	[74]
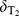	1	[69]	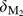	0.09	[29]
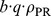	0.67	[29]	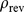	0.5	[31]
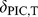	0.35	[21], [75]	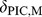	0.0035	[29]
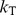	0.35	[31]	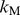	0.07	[29]
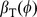	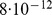	[76]	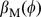	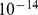	[29]
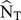	1000	[74]	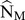	100	[74]
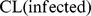	23	[70]	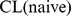	2.3	[71], [72]

All parameters refer to the absence of drug treatment 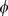. All parameters in units [1/day], except 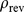 and 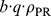 (unit less). 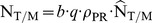 [29].

The presented modeling approach may be extended to e.g. assess the consequences of TDF-based PrEP intervention on drug resistance emergence, or TDF-based PrEP efficacy in the case when resistant virus becomes transmitted. Also, the combined effects of emtricitabine (FTC) and TDF remain to be elucidated, but can be studied by extending the presented model with the pharmacokinetics of FTC, once data on intracellular FTC-triphosphate becomes available.

## Supporting Information

Table S1 **Assessment of alternative models for plasma TFV pharmacokinetics.** Goodness-of-fit in terms of the weighted residual sum of squared errors (WRSE) of model predicted vs. experimental data following either doses of 75, 150, 300 or 600 mg oral TDF from three independent clinical trials [7], [9], [12] for a one compartment- vs. a two compartment model. The models were compared by computing the Akaike information (AIC) and the model with the lowest AIC value was used subsequently (the two compartment model). Goodness-of-fit plots are shown in Fig. 2A-B (main article).
(PDF)

Table S2
**Predicted individual TFV-DP elimination kinetics.** Estimated individual plateau concentrations 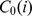 and elimination rates 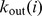 of TFV-DP from PBMCs (after treatment cessation). Parameters were estimated assuming first-order decay kinetics according to: 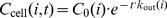 using the data from [11].(PDF)

Table S3 **Contingency table for infection events during once daily PrEP with 300 mg TDF.** Predictions are based on 2000 ‘virtual patients’ simulations respectively. The first number in the brackets in columns 2–6 indicates the number of ‘virtual patients’ that remained uninfected after viral challenge, whereas the second number indicates the number of patients that became infected. For example, when 20% of once daily 300 mg TDF pills are ingested and patients are challenged with inoculum size one (one virus reaches a target cell environment), 1927 ‘virtual patients’ remain uninfected, whereas 73 became infected.
(PDF)

Table S4 **Contingency table of infection events for one week of TDF-based PrEP with 300 mg started around the time of exposure (1w-PrEP/PEP).** Predictions are based on 2000 ‘virtual patients’ simulations respectively. The first number in the brackets in columns 2–6 indicates the number of ‘virtual patients’ that remained uninfected after viral challenge, whereas the second number indicates the number of patients that became infected. For example, when 300 mg TDF is taken 6 hours before viral challenge, continued for one week and patients are challenged with inoculum size one (one virus reaches a target cell environment), 1866 ‘virtual patients’ remain uninfected, whereas 134 became infected.
(PDF)

Table S5 **Contingency table of infection events for a single oral TDF dose 300 mg (sd-PrEP).** Predictions are based on 2000 ‘virtual patients’ simulations respectively. The first number in the brackets in columns 2–6 indicates the number of ‘virtual patients’ that remained uninfected after viral challenge, whereas the second number indicates the number of patients that became infected. For example, when 300 mg TDF are taken 1 hour before viral challenge and patients are challenged with inoculum size one (one virus reaches a target cell environment), 1818 virtual patients remain uninfected, whereas 182 became infected.
(PDF)

Table S6
**Contingency table of infection events for a single oral TDF dose 600mg (sd-PrEP).** Predictions are based on 2000 ‘virtual patients’ simulations respectively. The first number in the brackets in columns 2–6 indicates the number of ‘virtual patients’ that remained uninfected after viral challenge, whereas the second number indicates the number of patients that became infected. For example, when 600mg TDF are taken 1hour before viral challenge and patients are challenged with inoculum size one (one virus reaches a target cell environment), 1839 virtual patients remain uninfected, whereas 161 became infected. 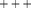 Inoculum size has a significant impact on the number of infections at the p
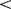
0.01 level (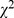-test).(PDF)

Table S7
**Statistical test of difference of prophylactic efficacy between 300 mg sd-PrEP and 600 mg sd-PrEP with TDF.** The distinct fields show the p-value for a 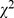-test between the prophylactic efficacy between 300 mg and 600 mg sd-PrEP with TDF. The predicted outcome was significantly different between the two distinct dosing regimens, if the p-value is p
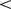
0.05, or p
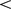
0.01 respectively (yellow- and red-shaded fields).(PDF)

Text S1 **The supplementary text contains the derivation of the model for intracellular TFV-DP uptake and anabolism as well as a model evaluation.**
(PDF)

Text S2 **The supplementary text contains the derivation of an approximate analytical formula for the computation of the probability of infection with distinct virus inoculum sizes in relation to the concentration of TFV-DP present.**
(PDF)
