# Correction: Pharmacokinetics and Pharmacodynamics of the Reverse Transcriptase Inhibitor Tenofovir and Prophylactic Efficacy against HIV-1 Infection

**DOI:** 10.1371/annotation/fb73d0f4-1cd8-481d-bddd-20439896102a

**Published:** 2012-11-07

**Authors:** Sulav Duwal, Christof Schütte, Max von Kleist

There are errors in Table 1. The correct table can be found here: 

**Figure:**
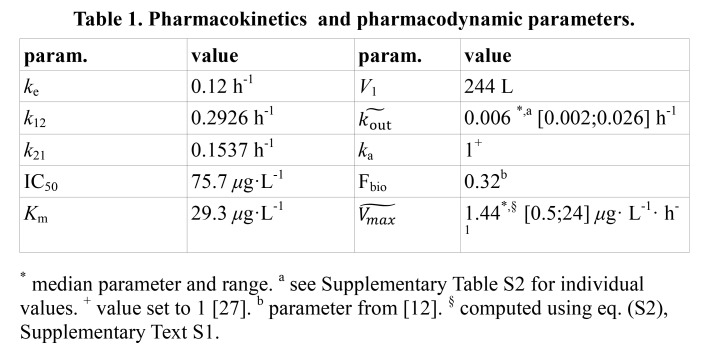


.

