# Supplementary material for: Pharmacokinetics and Pharmacodynamics of the Reverse Transcriptase Inhibitor Tenofovir and Prophylactic Efficacy against HIV-1 Infection
Source: PLoS One. 2012 Jul 11;7(7):e40382. doi: 10.1371/journal.pone.0040382 (PMC3394807; doi:10.1371/journal.pone.0040382)
Supplement: Table S1 — Assessment of alternative models for plasma TFV pharmacokinetics. Goodness-of-fit in terms of the weighted residual sum of squared errors (WRSE) of model predicted vs. experimental data following either doses of 75, 150, 300 or 600 mg oral TDF from three independent clinical trials [7], [9], [12] for a one compartment- vs. a two compartment model. The models were compared by computing the Akaike information (AIC) and the model with the lowest AIC value was used subsequently (the two compartment model). Goodness-of-fit plots are shown in Fig. 2A-B (main article). (PDF) [file pone.0040382.s001.pdf]

Table S1. Assessment of alternative models for plasma TFV pharmacokinetics.

	One Compartment Model	Two Compartment Model
WRSE*	8.54	6.24
AIC $^\flat$	87.63	79.41

Goodness of fit in terms of the weighted residual sum of squared errors (WRSE) of model predicted vs. experimental data following either doses of 75, 150, 300 or 600mg oral TDF from three independent clinical trials [1–3] for a one compartment- vs. a two compartment model. The models were compared by computing the Akaike information (AIC) and the model with the lowest AIC value was used subsequently (the two compartment model). Goodness-of-fit plots are shown in Fig. 2A-B (main article). * computed using eq. (1) (main article). $^\flat$ computed using eq. (2) (main article).

References

1. Barditch-Crovo P, Deeks SG, Collier A, Safrin S, Coakley DF, et al. (2001) Phase I/II trial of the pharmacokinetics, safety, and antiretroviral activity of tenofovir disoproxil fumarate in human immunodeficiency virus-infected adults. Antimicrob Agents Chemother 45: 2733–2739.

2. Chittick GE, Zong J, Blum MR, Sorbel JJ, Begley JA, et al. (2006) Pharmacokinetics of tenofovir disoproxil fumarate and ritonavir-boosted saquinavir mesylate administered alone or in combination at steady state. Antimicrob Agents Chemother 50: 1304–1310.

3. Droste JAH, van Wissen CPWGMV, Kearney BP, Buffels R, Vanhorssen PJ, et al. (2005) Pharmacokinetic study of tenofovir disoproxil fumarate combined with rifampin in healthy volunteers. Antimicrob Agents Chemother 49: 680–684.
