# Supplementary material for: Pharmacokinetics and Pharmacodynamics of the Reverse Transcriptase Inhibitor Tenofovir and Prophylactic Efficacy against HIV-1 Infection
Source: PLoS One. 2012 Jul 11;7(7):e40382. doi: 10.1371/journal.pone.0040382 (PMC3394807; doi:10.1371/journal.pone.0040382)
Supplement: Table S2 — Predicted individual TFV-DP elimination kinetics. Estimated individual plateau concentrations and elimination rates of TFV-DP from PBMCs (after treatment cessation). Parameters were estimated assuming first-order decay kinetics according to: using the data from [11]. (PDF) [file pone.0040382.s002.pdf]

Table S2. Predicted individual TFV-DP elimination kinetics.

Pat.#	$C_0(i)$ (fMol/10^6 cells)	$k_{\text{out}}(i)$ (h^{-1})	$t^{\flat}_{1/2}$ (h)
1	80.02	0.0054	129.2
2	91.21	0.0018	386.2
3	241	0.026	26.7
4	144.1	0.0023	296.9
5	61.33	0.0035	196.5
6	111.5	0.0064	109.1
7	338.6	0.023	29.7
8	75.61	0.0068	101.4
mean (± std)	143 (± 98)	0.0094 (± 0.0096)	159.4 (± 127)
weighted mean (± std)*	156 (± 166)	0.012 (± 0.01)	125.5 (± 134)
median (range)	101 (61;339)	0.0059 (0.002;0.026)	119.1 (26.7;386.2)

Estimated individual plateau concentrations $C_0(i)$ and elimination rates $k_{\text{out}}(i)$ of TFV-DP from PBMCs (after treatment cessation). Parameters were estimated assuming first-order decay kinetics according to: $C_{\text{cell}}(i,t) = C_0(i) \cdot e^{-t \cdot k_{\text{out}}(i)}$ using the data from [1]. * weighted mean $= \frac{1}{\sum_i R^2(i)} \cdot \sum_i R^2(i) \cdot x(i)$, where $x(i)$ is either $C_0(i)$, $k_{\text{out}}(i)$ or $t_{1/2}(i)$ and $R^2(i)$ is the pearson's correlation coefficient between experimental and predicted decay. $^\flat$ Halflife computed using $t_{1/2} = \ln(2)/k_{\text{out}}(i)$.

References

1. Hawkins T, Veikley W, Claire RLS, Guyer B, Clark N, et al. (2005) Intracellular pharmacokinetics of tenofovir diphosphate, carbovir triphosphate, and lamivudine triphosphate in patients receiving triple-nucleoside regimens. J Acquir Immune Defic Syndr 39: 406–411.
