# Supplementary material for: Pharmacokinetics and Pharmacodynamics of the Reverse Transcriptase Inhibitor Tenofovir and Prophylactic Efficacy against HIV-1 Infection
Source: PLoS One. 2012 Jul 11;7(7):e40382. doi: 10.1371/journal.pone.0040382 (PMC3394807; doi:10.1371/journal.pone.0040382)
Supplement: Table S3 — Contingency table for infection events during once daily PrEP with 300 mg TDF. Predictions are based on 2000 ‘virtual patients’ simulations respectively. The first number in the brackets in columns 2–6 indicates the number of ‘virtual patients’ that remained uninfected after viral challenge, whereas the second number indicates the number of patients that became infected. For example, when 20% of once daily 300 mg TDF pills are ingested and patients are challenged with inoculum size one (one virus reaches a target cell environment), 1927 ‘virtual patients’ remain uninfected, whereas 73 became infected. (PDF) [file pone.0040382.s003.pdf]

Table S3. Contingency table for infection events during once daily PrEP with 300mg TDF

	Adherence				
Inoc. size	20%	40%	60%	80%	100%
1	(1927;73)	(1954;46)	(1959;41)	(1953;47)	(1952;48)
5	(1668;332)	(1720;280)	(1755;245)	(1768;232)	(1794;206)
20	(1046;954)	(1170;830)	(1274;726)	(1287;713)	(1323;677)
100	(154;1846)	(255;1745)	(312;1688)	(346;1654)	(337;1663)
	$p < 0.01^{+++}$	$p < 0.01^{+++}$	$p < 0.01^{+++}$	$p < 0.01^{+++}$	$p < 0.01^{+++}$

Predictions are based on 2000 'virtual patients' simulations respectively. The first number in the brackets in columns 2-6 indicates the number of 'virtual patients' that remained uninfected after viral challenge, whereas the second number indicates the number of patients that became infected. For example when 20% of once daily 300mg TDF pills are ingested and patients are challenged with inoculum size one (one virus reaches a target cell environment) 1927 'virtual patients' remained uninfected, whereas 73 became infected.
$^{+++}$ Inoculum size has a significant impact on the number of infections at the $p < 0.01$ level (χ^2-test).
