# Supplementary material for: Pharmacokinetics and Pharmacodynamics of the Reverse Transcriptase Inhibitor Tenofovir and Prophylactic Efficacy against HIV-1 Infection
Source: PLoS One. 2012 Jul 11;7(7):e40382. doi: 10.1371/journal.pone.0040382 (PMC3394807; doi:10.1371/journal.pone.0040382)
Supplement: Table S4 — Contingency table of infection events for one week of TDF-based PrEP with 300 mg started around the time of exposure (1w-PrEP/PEP). Predictions are based on 2000 ‘virtual patients’ simulations respectively. The first number in the brackets in columns 2–6 indicates the number of ‘virtual patients’ that remained uninfected after viral challenge, whereas the second number indicates the number of patients that became infected. For example, when 300 mg TDF is taken 6 hours before viral challenge, continued for one week and patients are challenged with inoculum size one (one virus reaches a target cell environment), 1866 ‘virtual patients’ remain uninfected, whereas 134 became infected. (PDF) [file pone.0040382.s004.pdf]

Table S4. Contingency table of infection events for one week of TDF-based PrEP with 300mg started around the time of exposure (1w-PrEP/PEP).

	Time lapse between drug intake and viral exposure				
	6hr before	1hr before	1hr after	6hr after	48hr after
1	(1866;134)	(1850;150)	(1850;150)	(1831;169)	(1813;187)
5	(1393;607)	(1338;662)	(1290;710)	(1260;740)	(1213;787)
20	(537;1463)	(408;1592)	(354;1646)	(289;1711)	(252;1748)
100	(26;1974)	(4;1996)	(3;1997)	(0;2000)	(0;2000)
	$p < 0.01^{+++}$	$p < 0.01^{+++}$	$p < 0.01^{+++}$	$p < 0.01^{+++}$	$p < 0.01^{+++}$

Predictions are based on 2000 'virtual patients' simulations respectively. The first number in the brackets in columns 2-6 indicates the number of 'virtual patients' that remained uninfected after viral challenge, whereas the second number indicates the number of patients that became infected. For example, when 300mg TDF is taken 6 hours before viral challenge, continued for one week and patients are challenged with inoculum size one (one virus reaches a target cell environment), 1866 'virtual patients' remained uninfected, whereas 134 became infected. $^{+++}$ Inoculum size has a significant impact on the number of infections at the $p < 0.01$ level (χ^2-test).
