# Supplementary material for: Pharmacokinetics and Pharmacodynamics of the Reverse Transcriptase Inhibitor Tenofovir and Prophylactic Efficacy against HIV-1 Infection
Source: PLoS One. 2012 Jul 11;7(7):e40382. doi: 10.1371/journal.pone.0040382 (PMC3394807; doi:10.1371/journal.pone.0040382)
Supplement: Table S5 — Contingency table of infection events for a single oral TDF dose 300 mg (sd-PrEP). Predictions are based on 2000 ‘virtual patients’ simulations respectively. The first number in the brackets in columns 2–6 indicates the number of ‘virtual patients’ that remained uninfected after viral challenge, whereas the second number indicates the number of patients that became infected. For example, when 300 mg TDF are taken 1 hour before viral challenge and patients are challenged with inoculum size one (one virus reaches a target cell environment), 1818 virtual patients remain uninfected, whereas 182 became infected. (PDF) [file pone.0040382.s005.pdf]

Table S5. Contingency table of infection events for a single oral TDF dose 300mg (sd-PrEP).

	Drug intake prior to viral exposure				
Inoc. size	1 hr	6 hr	12 hr	24 hr	48 hr
1	(1818;182)	(1863;137)	(1863;137)	(1900;100)	(1873;127)
5	(1318;682)	(1383;617)	(1403;597)	(1422;578)	(1488;512)
20	(388;1612)	(497;1503)	(617;1383)	(600;1400)	(706;1294)
100	(3;1997)	(18;1982)	(46;1954)	(73;1927)	(80;1920)
	$p < 0.01^{+++}$	$p < 0.01^{+++}$	$p < 0.01^{+++}$	$p < 0.01^{+++}$	$p < 0.01^{+++}$

Predictions are based on 2000 'virtual patients' simulations respectively. The first number in the brackets in columns 2-6 indicates the number of 'virtual patients' that remained uninfected after viral challenge, whereas the second number indicates the number of patients that became infected. For example, when 300mg TDF are taken 1hour before viral challenge and patients are challenged with inoculum size one (one virus reaches a target cell environment), 1818 virtual patients remain uninfected, whereas 182 became infected. $^{+++}$ Inoculum size has a significant impact on the number of infections at the p < 0.01 level (χ^2-test).
