# Supplementary material for: Pharmacokinetics and Pharmacodynamics of the Reverse Transcriptase Inhibitor Tenofovir and Prophylactic Efficacy against HIV-1 Infection
Source: PLoS One. 2012 Jul 11;7(7):e40382. doi: 10.1371/journal.pone.0040382 (PMC3394807; doi:10.1371/journal.pone.0040382)
Supplement: Table S7 — Statistical test of difference of prophylactic efficacy between 300 mg sd-PrEP and 600 mg sd-PrEP with TDF. The distinct fields show the p-value for a -test between the prophylactic efficacy between 300 mg and 600 mg sd-PrEP with TDF. The predicted outcome was significantly different between the two distinct dosing regimens, if the p-value is p0.05, or p0.01 respectively (yellow- and red-shaded fields). (PDF) [file pone.0040382.s007.pdf]

Table S7. Statistical test of difference of prophylactic efficacy between 300mg sd-PrEP and 600mg sd-PrEP with TDF.

	Drug intake prior to viral exposure				
Inoc. size	1 hr	6 hr	12 hr	24 hr	48 hr
1	$p = 0.26$	$p = 0.19$	$p = 0.76$	$p = 0.3$	$p = 0.6$
5	$p < 0.05$	$p = 0.3$	$p = 0.53$	$p < 0.05$	$p = 0.4$
20	$p = 0.2$	$p = 0.16$	$p = 0.47$	$p < 0.01$	$p = 0.26$
100	$p = 0.2$	$p = 0.18$	$p = 0.2$	$p < 0.01$	$p < 0.05$

The distinct fields show the p-value for a χ^2-test between the prophylactic efficacy between 300mg and 600mg sd-PrEP with TDF. The predicted outcome was significantly different between the two distinct dosing regimens, if the p-value is $p<0.05$, or $p<0.01$ respectively (yellow- and red-shaded fields).
