# Supplementary material for: Pharmacokinetics and Pharmacodynamics of the Reverse Transcriptase Inhibitor Tenofovir and Prophylactic Efficacy against HIV-1 Infection
Source: PLoS One. 2012 Jul 11;7(7):e40382. doi: 10.1371/journal.pone.0040382 (PMC3394807; doi:10.1371/journal.pone.0040382)
Supplement: Text S1 — The supplementary text contains the derivation of the model for intracellular TFV-DP uptake and anabolism as well as a model evaluation. (PDF) [file pone.0040382.s008.pdf]

S1 Supplementary Text

Kinetics of Tenofovir Uptake & Anabolism in Target Cells

After cellular uptake, tenofovir requires two subsequent phorphorylation steps for activation. The cellular uptake and anabolism (phosphorylization) scheme is shown below.

$$\mathrm{TFV_p} \rightleftharpoons \mathrm{TFV_c} \rightleftharpoons \text{TFV-MP} \rightleftharpoons \text{TFV-DP}$$

where $\mathrm{TFV_p}$ and $\mathrm{TFV_c}$ denote the concentration of TFV in the blood plasma and cellular space respectively and TFV-MP and TFV-DP denote the intracellular mono- and di-phosphorylated TFV. Due to the lack of detailed *in vivo* studies regarding concentration time profiles of the distinct intermediate forms, a modular model for intracellular pharmacokinetics of all intermediate forms cannot be justified. As often practiced in kinetic studies, the processes stated above were therefore subsumed by one compartment with 'lumped' parameters instead of 3 compartments with more parameters. In the sequel, we will refer to plasma concentration of TFV by C_1 and to the active moity by C_{cell}, consistent with the notation in the main manuscript.

Michaelis-Menten kinetics were used to model the uptake of tenofovir and its conversion to tenofovir diphosphate (TFV-DP) as shown in eq. (S1) below:

$$\frac{\mathrm{d}}{\mathrm{d}t}C_{\mathrm{cell}}(t,i) = \underbrace{\frac{V_{\max}(i)\cdot C_1}{K_{\mathrm{m}}+C_1}}_{\text{Michaelis Menten kinetics}} - \underbrace{C_{\mathrm{cell}}(t,i)\cdot k_{\mathrm{out}}(i)}_{\text{First order elimination of TFV-DP}} \quad , \tag{S1}$$

where $V_{\max}$ is the maximum reaction rate and K_{m} denotes the Michaelis-Menten constant. The parameter k_{out} is the first order elimination rate constant for TFV-DP (estimated in Supplementary Table S2). Under steady state assumptions, the equation can be solved for the $V_{\max}$ parameter.

$$V_{\max}(i) = C_{\mathrm{cell}}(i)\cdot k_{\mathrm{out}}(i)\cdot\left(\frac{K_{\mathrm{m}}}{C_1}+1\right) \tag{S2}$$

Thus, for assessing the kinetics of TFV-DP uptake & anabolism, only K_{m} needs to be estimated. Different types of kinetics for tenofovir anabolism in target cells can be achieved by varying K_{m} in relation to C_1 in eq. (S2). Two extreme cases, complete saturation and no saturation (linear kinetics), can be obtained by setting $K_{\mathrm{m}} << C_1$ and $K_{\mathrm{m}} >> C_1$ respectively.

Parameter Estimation and Model Assessment

In absence of *in vivo* data regarding the cellular uptake and anabolism of TFV-DP, pharmacokinetic parameters cannot be estimated directly. However, after coupling the pharmacokinetic model to the viral dynamics model proposed in eqs. (7)-(10) (main article) it is possible to predict viral decay kinetics and compare with clinical data for four dose regimens (75-, 150-, 300- and 600mg oral TDF) [1]. For the virus dynamics model, all parameters are known from the literature (see Table 2, main article), except for the pharmacokinetic-pharmacodynamic coupling parameter IC_{50} (fifty percent inhibitory TFV-DP concentration). Using this approach, parameter estimation (K_{m} and IC_{50}) and model selection allows to reverse-engineer the TFV anabolism & uptake kinetics.

In the sequel, we systematically implemented two different approaches to estimate viral load decay: (i) we estimated viral decay kinetics using population estimates (weighted mean) for the intracellular elimination of TFV-DP k_{out} (see Supplementary Table S2), ignoring inter-individual differences in the cellular kinetics of TFV-DP. (ii) We estimated viral decay kinetics using individual intracellular elimination kinetics of TFV-DP $k_{\text{out}}(\text{i})$ (see Supplementary Table S2), explicitly considering inter-individual differences in the cellular kinetics of TFV-DP.

During the dose escalation study [1], subjects received either 75- 150- 300 or 600mg oral TDF for 28 consecutive days and were monitored for dosing period and the following 28 days. The estimated parameters and goodness-of-fit measures for the two different models are given below in Table A1.

Table A1. Estimated parameters and goodness-of-fit for two alternative models

TFV-DP kinetics	pop. average	individual
IC_{50} μg/L	138.7	75.7
K_{m} μg/L	0.32	29.3
WRSE (75mg)	8.34	3.3
WRSE (150mg)	2.35	2.2
WRSE (300mg)	3.63	3.48
WRSE (600mg)	2.75	3.33
aggregate WRSE	17.07	12.30
AIC	104.88	94.36

Estimated parameters (IC_{50}, K_{m}) for a model using average TFV-DP intracellular kinetics (second column) and individual intracellular TFV-DP kinetics (third column). The bottom row indicates the AIC value with regard to predicting viral decay for four dosing regimens (75-, 150-, 300- and 600mg) [1]. Subsequently the model with the individual intracellular TFV-DP kinetics was chosen (third column). Model predicted vs. clinically observed viral decay kinetics are shown in Fig. 3 (main article).

References

1. Barditch-Crovo P, Deeks SG, Collier A, Safrin S, Coakley DF, et al. (2001) Phase I/II trial of the pharmacokinetics, safety, and antiretroviral activity of tenofovir disoproxil fumarate in human immunodeficiency virus-infected adults. Antimicrob Agents Chemother 45: 2733–2739.
