# Supplementary material for: Pharmacokinetics and Pharmacodynamics of the Reverse Transcriptase Inhibitor Tenofovir and Prophylactic Efficacy against HIV-1 Infection
Source: PLoS One. 2012 Jul 11;7(7):e40382. doi: 10.1371/journal.pone.0040382 (PMC3394807; doi:10.1371/journal.pone.0040382)
Supplement: Text S2 — The supplementary text contains the derivation of an approximate analytical formula for the computation of the probability of infection with distinct virus inoculum sizes in relation to the concentration of TFV-DP present. (PDF) [file pone.0040382.s009.pdf]

S2 Supplementary Text

Probability of Infection

In the sequel, we will derive an approximate analytical solution for computing the probability of HIV-1 infection P_{inf} based on the mathematical model used in the main manuscript. The analytical approximation will be compared with simulation results.

From probability conservation, it follows that

$$P_{\text{inf}} = 1 - P_{\emptyset} \tag{S1}$$

where $P_{\emptyset}$ is the probability that the infection is prevented (virus extinction). The process of virus extinction can be viewed as a branching process, where the virus may be entirely cleared during each consecutive replication cycle $\tau = 1 ... \infty$.

$$P_{\emptyset} = \underbrace{P\big(\emptyset | V_0(\tau = 1) = n\big)}_{\text{clearance during first repl. cycle}} + \underbrace{\sum_{\tau=2}^{\infty} \sum_{i=1}^{\infty} P\big(\emptyset | V_0(\tau) = i\big) \cdot P\big(V_0(\tau) = i\big)}_{\text{clearance during subsequent repl. cycles}}. \tag{S2}$$

where $P(V_0(\tau) = i)$ denotes the probability to have $i = 1 ... \infty$ infectious viruses at the beginning of the τth infection cycle, and $P(\emptyset | V_0(\tau) = i)$ denotes the conditional probability that the infection becomes cleared during the τth infection cycle when the number of infectious virus was i at the beginning of the τth infection cycle. In our case, we want to know the infection probability for specific inoculum sizes at $\tau = 1$. From eq. (S2) it follows that

$$P\big(\emptyset | V_0(\tau = 1) = n\big) \leq P_{\emptyset}.$$

Clearance of HIV infection may be assumed to be more likely as long as the number of viral particles is low. Since virus released by late infected cells (T_2, M_2) results on average in an amplification of factor 100 (replication within macrophages) to 1000 (replication within T-cells) [1], it can be assumed that the probability of clearance during the first replication cycle is much larger than the probability of clearance during subsequent replication cycles, i.e. $P\big(\emptyset | V_0(\tau = 1) = n\big) \gg \sum_{\tau=2}^{\infty} \sum_{i=1}^{\infty} P\big(\emptyset | V_0(\tau) = i\big) \cdot P\big(V_0(\tau) = i\big)$. It therefore follows that $P_{\emptyset}$ may be approximated only by predicting the probability of clearance during the first round of replication:

$$\begin{aligned} P_{\emptyset} &= P(\emptyset | V_0 = n) + \varepsilon \\ \Rightarrow P_{\emptyset} &\approx P(\emptyset | V_0 = n) \end{aligned} \tag{S3}$$

For ease of notation we have replaced $V_0(\tau = 1)$ by V_0. Under the reasonable assumption that virus challenges are independent events, we may further write

$$P_{\emptyset} \approx P(\emptyset | V_0 = 1)^n \tag{S4}$$

It now follows from eq. (S1), (S3) & (S4) that the infection (proliferation) probability in the context of n inoculated viral particles is computed according to

$$P(\text{inf} | V_0 = n) \approx 1 - P(\emptyset | V_0 = n) = 1 - (P(\emptyset | V_0 = 1))^n \tag{S5}$$

Therefore, an analytical expression for the probability of non-infection (virus clearance) within the first round of replication with inoculum size one $P(\emptyset | V_0 = 1)$ needs to be derived in order to compute the infection probability with an arbitrary inoculum size i analytically (see below for derivation). The % infections prevented can then be computed using eq. (11) (main article) by considering the two scenarios of drug presence and -absence.

Analytical Solution for the Probability of non-Infection

As previously described, we are seeking an analytical expression for the probability of virus clearance during the first replication cycle in the case when one virus particle was inoculated $P(\emptyset|\mathrm{V}_0 = 1)$. The viral replication cycle can be interpreted as a branching process, see Fig. S1 (left panel), where the infection may be prevented during each stage $\{V_0, \mathrm{T}_1, \mathrm{T}_2, \mathrm{M}_1$ and $\mathrm{M}_2\} \rightarrow \emptyset$ before the production of viral progeny. We may thus write:

$$\begin{aligned} P(\emptyset|V_0 = 1) &= P(\mathrm{V}_0 \rightarrow \emptyset) \\ &+ P(\mathrm{V}_0 \rightarrow \mathrm{T}_1 \rightarrow \emptyset) \\ &+ P(\mathrm{V}_0 \rightarrow \mathrm{M}_1 \rightarrow \emptyset) \\ &+ P(\mathrm{V}_0 \rightarrow \mathrm{T}_1 \rightarrow \mathrm{T}_2 \rightarrow \emptyset) \\ &+ P(\mathrm{V}_0 \rightarrow \mathrm{M}_1 \rightarrow \mathrm{M}_2 \rightarrow \emptyset), \end{aligned} \tag{S6}$$

where $P(\mathrm{V}_0 \rightarrow \emptyset)$ denotes the probability that free virus becomes entirely cleared before infecting cells and e.g. $P(\mathrm{V}_0 \rightarrow \mathrm{T}_1 \rightarrow \mathrm{T}_2 \rightarrow \emptyset)$ denotes the joint probability of extinction in the late infected T-cell compartment ($\mathrm{T}_2 \rightarrow \emptyset$) before the production of viral progeny and after successful infection and integration into T-cells ($\mathrm{V}_0 \rightarrow \mathrm{T}_1 \rightarrow \mathrm{T}_2$). The joint probabilities of extinction in infectious compartments $\mathrm{T}_1, \mathrm{T}_2, \mathrm{M}_1$ and M_2 can be computed from the conditional probabilities of extinction:

$$\begin{aligned} P(\mathrm{V}_0 \rightarrow \mathrm{T}_1 \rightarrow \emptyset) &= P(\mathrm{T}_1 \rightarrow \emptyset|\mathrm{V}_0 \rightarrow \mathrm{T}_1) \cdot P(\mathrm{V}_0 \rightarrow \mathrm{T}_1) \\ P(\mathrm{V}_0 \rightarrow \mathrm{M}_1 \rightarrow \emptyset) &= P(\mathrm{M}_1 \rightarrow \emptyset|\mathrm{V}_0 \rightarrow \mathrm{M}_1) \cdot P(\mathrm{V}_0 \rightarrow \mathrm{M}_1) \\ P(\mathrm{V}_0 \rightarrow \mathrm{T}_1 \rightarrow \mathrm{T}_2 \rightarrow \emptyset) &= P(\mathrm{T}_2 \rightarrow \emptyset|\mathrm{T}_1 \rightarrow \mathrm{T}_2) \cdot P(\mathrm{T}_1 \rightarrow \mathrm{T}_2|\mathrm{V}_0 \rightarrow \mathrm{T}_1) \cdot P(\mathrm{V}_0 \rightarrow \mathrm{T}_1) \\ P(\mathrm{V}_0 \rightarrow \mathrm{M}_1 \rightarrow \mathrm{M}_2 \rightarrow \emptyset) &= P(\mathrm{M}_2 \rightarrow \emptyset|\mathrm{M}_1 \rightarrow \mathrm{M}_2) \cdot P(\mathrm{M}_1 \rightarrow \mathrm{M}_2|\mathrm{V}_0 \rightarrow \mathrm{M}_1) \cdot P(\mathrm{V}_0 \rightarrow \mathrm{M}_1), \end{aligned} \tag{S7}$$

which are related to the reaction rates of the utilized model (see Fig. 1B and Table 2, main manuscript) via:

$$\begin{aligned} P(\mathrm{V}_0 \rightarrow \emptyset) &= \frac{\mathrm{cl_V}}{\beta_\mathrm{T}(\mathrm{t}) \cdot \mathrm{T_U} + \beta_\mathrm{M}(\mathrm{t}) \cdot \mathrm{M_U} + \mathrm{cl_V}} \\ P(\mathrm{V}_0 \rightarrow \mathrm{T}_1) &= \frac{\beta_\mathrm{T}(\mathrm{t}) \cdot \mathrm{T_U}}{\beta_\mathrm{T}(\mathrm{t}) \cdot \mathrm{T_U} + \beta_\mathrm{M}(\mathrm{t}) \cdot \mathrm{M_U} + \mathrm{cl_V}} \\ P(\mathrm{V}_0 \rightarrow \mathrm{M}_1) &= \frac{\beta_\mathrm{M}(\mathrm{t}) \cdot \mathrm{M_U}}{\beta_\mathrm{T}(\mathrm{t}) \cdot \mathrm{T_U} + \beta_\mathrm{M}(\mathrm{t}) \cdot \mathrm{M_U} + \mathrm{cl_V}} \\ P(\mathrm{T}_1 \rightarrow \emptyset|\mathrm{V}_0 \rightarrow \mathrm{T}_1) &= \frac{\delta_\mathrm{T1} + \delta_\mathrm{PIC,T}}{\delta_\mathrm{T1} + \delta_\mathrm{PIC,T} + k_\mathrm{T}} \\ P(\mathrm{M}_1 \rightarrow \emptyset|\mathrm{V}_0 \rightarrow \mathrm{M}_1) &= \frac{\delta_\mathrm{M1} + \delta_\mathrm{PIC,M}}{\delta_\mathrm{M1} + \delta_\mathrm{PIC,M} + k_\mathrm{M}} \\ P(\mathrm{T}_1 \rightarrow \mathrm{T}_2|\mathrm{V}_0 \rightarrow \mathrm{T}_1) &= \frac{k_\mathrm{T}}{\delta_\mathrm{T1} + \delta_\mathrm{PIC,T} + k_\mathrm{T}} \\ P(\mathrm{M}_1 \rightarrow \mathrm{M}_2|\mathrm{V}_0 \rightarrow \mathrm{M}_1) &= \frac{k_\mathrm{M}}{\delta_\mathrm{M1} + \delta_\mathrm{PIC,M} + k_\mathrm{M}} \\ P(\mathrm{T}_2 \rightarrow \emptyset|\mathrm{T}_1 \rightarrow \mathrm{T}_2) &= \frac{\delta_\mathrm{T2}}{\delta_\mathrm{T2} + \hat{\mathrm{N}}_\mathrm{T}} \\ P(\mathrm{M}_2 \rightarrow \emptyset|\mathrm{M}_1 \rightarrow \mathrm{M}_2) &= \frac{\delta_\mathrm{M2}}{\delta_\mathrm{M2} + \hat{\mathrm{N}}_\mathrm{M}}, \end{aligned} \tag{S8}$$

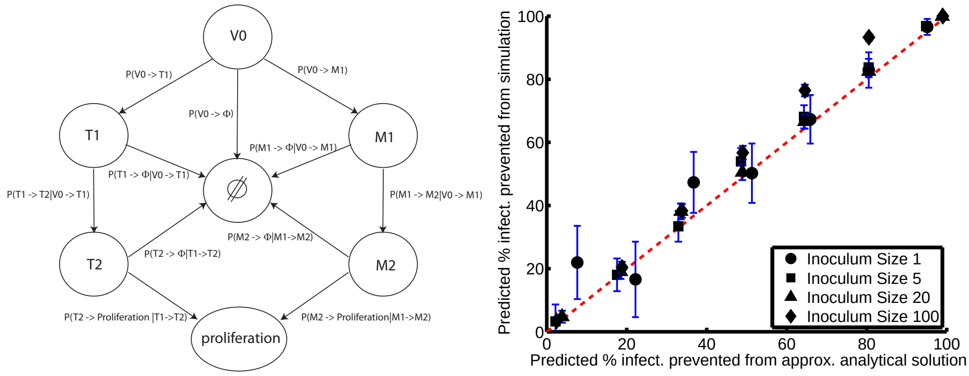

Figure S1. Depiction of viral replication cycle as a branching process and comparison of % infections prevented derived from analytical solution after simplification and after simulation of the entire model. Left panel: Depiction of viral replication cycle as a branching process with the respective conditional probabilities of viral extinction displayed on the arrows. The infection may be prevented during each stage before the production of viral progeny. Right Panel: Comparison of simulated % infections prevented (y-axis) and % infections prevented computed analytically after simplification (x-axis; computed using eq. (S5) in conjunction with eq. (11) in the main article). The blue vertical error bars indicate the empirical confidence range from the stochastic simulation (2000 stochastic runs for each TFV-DP concentration and inoculum size respectively).

with parameter $\mathrm{cl_V} = \mathrm{CL} + \mathrm{T_U} \cdot \mathrm{CL_T(t)} + \mathrm{M_U} \cdot \mathrm{CL_M(t)}$. All parameters are exemplified in the main manuscript and parameter values can be derived from Table 2 (main manuscript). Before the onset of infection, parameters $\mathrm{T_U}$ and $\mathrm{M_U}$ may be approximated by $\lambda_\mathrm{T}/\delta_\mathrm{T}$ and $\lambda_\mathrm{M}/\delta_\mathrm{M}$ respectively.

Note, that the parameters $\beta_\mathrm{T}(\mathrm{t}), \beta_\mathrm{M}(\mathrm{t}), \mathrm{CL_T(t)}$ and $\mathrm{CL_M(t)}$ are affected by the intracellular concentrations of TFV-DP via eq. (8)-(10) in the main manuscript. This allows us to assess the % infections prevented for arbitrary intracellular TFV-DP concentrations and arbitrary virus inoculum sizes via eqs. (S5), (S6)-(S8) in conjunction with eq. (11) (main article), which is shown in Fig. 6 (main article).

A comparison between the predicted % infections prevented resulting from this approximation and simulated % infections prevented using the entire model is shown in Fig. S1 (right panel) herein. As can be seen, the analytical solution (on the x-axis) may in some cases slightly underpredict the % infections prevented because it only considers the first round of replication (see eq. (S3)) as compared to the simulation of the full model. This potential error is, however, very minor and therefore we believe that the approximate analytical solution is reasonable to assess the % infections prevented in the context of our model.

References

1. Sedaghat AR, Dinoso JB, Shen L, Wilke CO, Siliciano RF (2008) Decay dynamics of HIV-1 depend on the inhibited stages of the viral life cycle. Proc Natl Acad Sci U S A 105: 4832–4837.
